# Developmental asynchrony and antagonism of sex determination pathways in a lizard with temperature-induced sex reversal

**DOI:** 10.1038/s41598-018-33170-y

**Published:** 2018-10-05

**Authors:** Sarah L. Whiteley, Vera Weisbecker, Arthur Georges, Arnault Roger Gaston Gauthier, Darryl L. Whitehead, Clare E. Holleley

**Affiliations:** 1 Institute for Applied Ecology, University of Canberra, Canberra, ACT Australia; 2 Australian National Wildlife Collection, National Research Collections Australia, CSIRO, Canberra, ACT Australia; 3 School of Biological Sciences, University of Queensland, Brisbane, QLD Australia; 4 School of Biomedical Sciences, University of Queensland, Brisbane, QLD Australia

## Abstract

Vertebrate sex differentiation follows a conserved suite of developmental events: the bipotential gonads differentiate and shortly thereafter sex specific traits become dimorphic. However, this may not apply to squamates, a diverse vertebrate lineage comprising of many species with thermosensitive sexual development. Of the three species with data on the relative timing of gonad differentiation and genital dimorphism, the females of two (*Niveoscincus ocellatus* and *Barisia imbricata*) exhibit a phase of temporary pseudohermaphroditism or TPH (gonads have differentiated well before genital dimorphism). We report a third example of TPH in *Pogona vitticeps*, an agamid with temperature-induced male to female sex reversal. These findings suggest that for female squamates, genital and gonad development may not be closely synchronised, so that TPH may be common. We further observed a high frequency of ovotestes, a usually rare gonadal phenotype characterised by a mix of male and female structures, exclusively associated with temperature-induced sex reversal. We propose that ovotestes are evidence of a period of antagonism between male and female sex-determining pathways during sex reversal. Female sexual development in squamates is considerably more complex than has been appreciated, providing numerous avenues for future exploration of the genetic and hormonal cues that govern sexual development.

## Introduction

Sex determination and differentiation in amniotes is widely accepted to follow a well-defined sequence^1–3^. Early in development, the bipotential gonads differentiate, then secrete sex-specific steroid hormones, which are thought to prompt the development of sex-specific traits, such as the male Wolffian or female Müllerian ducts (and regression of the opposing sex ducts), and the external genitalia (e.g. hemipenes/hemiclitores)^1,2,4–6^. Among reptiles the primary sex-determining cue can be either temperature or genetic^3^. In temperature-dependent sex determination (TSD), incubation temperature determines the sex of the individual during the thermosensitive period, which usually occurs in the middle-third of development^7,8^. The mechanism by which temperature influences sexual development in squamates (snakes and lizards) is not fully understood but is likely to involve epigenetic re-modelling via altered expression and/or splicing of chromatin modifying genes^9–11^. In contrast, gonadal differentiation is controlled in other squamates, by the presence, absence or dosage of as yet unidentified genes on sex chromosomes (genetic sex determination or GSD)^12–14^. Regardless of whether sex is controlled by TSD or GSD, the downstream molecular processes of gonad differentiation appear to be highly conserved^15–18^.

Although organisms tend to be classified as either TSD or GSD in the literature, in some species sex can be determined via gene–environment interactions^19,20^. This can occur when GSD is overridden by high or low incubation temperatures. In most cases of sex reversal in nature, the phenotype of the homogametic sex (ZZ or XX) becomes discordant with the sex chromosomes, though there are rare, mostly experimental examples of heterogametic (XY or ZW) sex reversal^3,21,22^. Such gene-environment interactions are possibly more common than assumed^20^, occurring in at least three squamate species - the spotted skink (*Niveoscincus ocellatus*), the three-lined skink (*Bassiana duperreyi*), and the central bearded dragon (*Pogona vitticeps*)^7,19,23^. The best-studied of these, *P. vitticeps*, exhibits GSD when eggs are incubated at moderate temperatures (ZW females, ZZm males)^24^. At high temperatures, ZZm males reverse their sex and develop as phenotypic females (ZZf herein). This process can lead to a complete transition from GSD to TSD within one generation^19^.

The sex determination system of *P. vitticeps* has provided novel insights into the molecular pathways underpinning the genetic and temperature influence of sex in reptiles, making this species an important emergent model organism supported by significant molecular resources^9,19,25,26^. A recent developmental study^27^ showed that body and genital development do not differ under TSD and GSD, but revealed an unexpected developmental trait: both genetic and temperature-induced females initially develop male genitalia (hemipenes), retain them for much of development, and then regress these structures close to hatching^27^.

**Figure 1:**
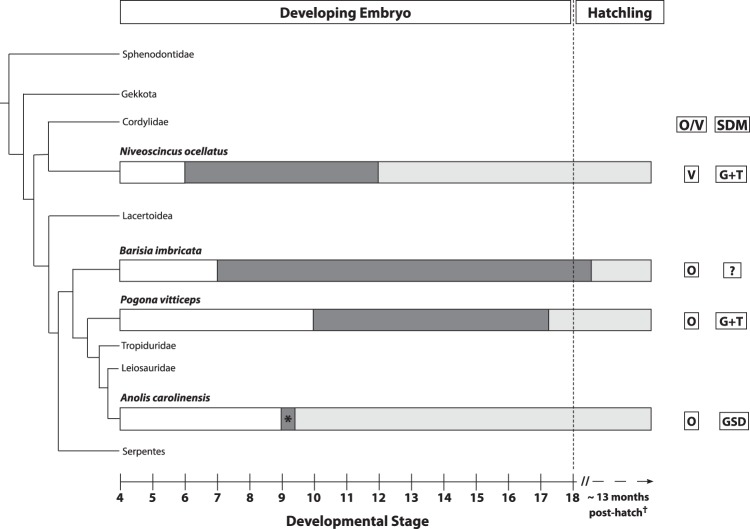
The timing and duration of temporary pseudohermaphroditism (TPH) in squamates. White bars indicate indeterminate sex, dark grey shows the TPH phase, and light grey indicates dimorphic sexes. The asterisk symbol for *Anolis carolinensis* indicates that there is a short delay (half a stage) between ovarian differentiation and genital dimorphism but is functionally lacking TPH. The dagger symbol on the dotted section of developmental stage axis indicates the approximate post-hatch timing of hemipenis regression in *Barisia imbricata*^31^. The timing of events are approximations standardised to the staging system described for *Pogona vitticeps*^27^. The reproductive mode (O/V) and sex determination mode (SDM) is reported for each species (O = oviparity, V = viviparity, G + T = genetic with thermal influence, GSD = genetic sex determination). The dashed vertical line at stage 18 denotes approximate time of hatching/birth. Phylogeny adapted from^70^, branch length is for illustrative purposes only.

The late development of female genitalia through regression of well-developed hemipenes is at odds with the general consensus that vertebrate gonad differentiation rapidly triggers sex-specific genital formation^6,28,29^. However, this assumption remains to be fully tested in squamates as there are only three studies examining the degree of synchronisation of gonad and genital development in this order. Of these examples, two phylogenetically disparate species (the imbricate alligator lizard, *Barisia imbricata*, and *N. ocellatus*; Fig. 1) display a developmental phase where differentiated ovaries exist alongside male genitalia across multiple developmental stages, which is a form of temporary pseudohermaphroditism (TPH)^30,31^. The third squamate species for which there are comparable developmental data, the Carolina anole (*Anolis carolinensis*), develops genitalia almost immediately after gonad differentiation in both males and females, so that TPH is functionally absent in this species^32,33^.

In this study, we demonstrate that sexual development of *P. vitticeps* is considerably more complex than expected based on current understanding of squamate sex determination and differentiation. In particular, we show histologically that asynchronous sexual development of the gonads and external genitalia of *P. vitticeps* under both normal and sex-reversing temperatures arises from a form of temporary pseudohermaphroditism, as in *B. imbricata* and *N. ocellatus*. We also show that temperature-induced sex reversal is characterised by the presence of ovotestes, a rare gonadal phenotype suggestive of a period of antagonism between genetic and thermal influences during sex determination.

## Results

### Timing of gonad differentiation

**Figure 2:**
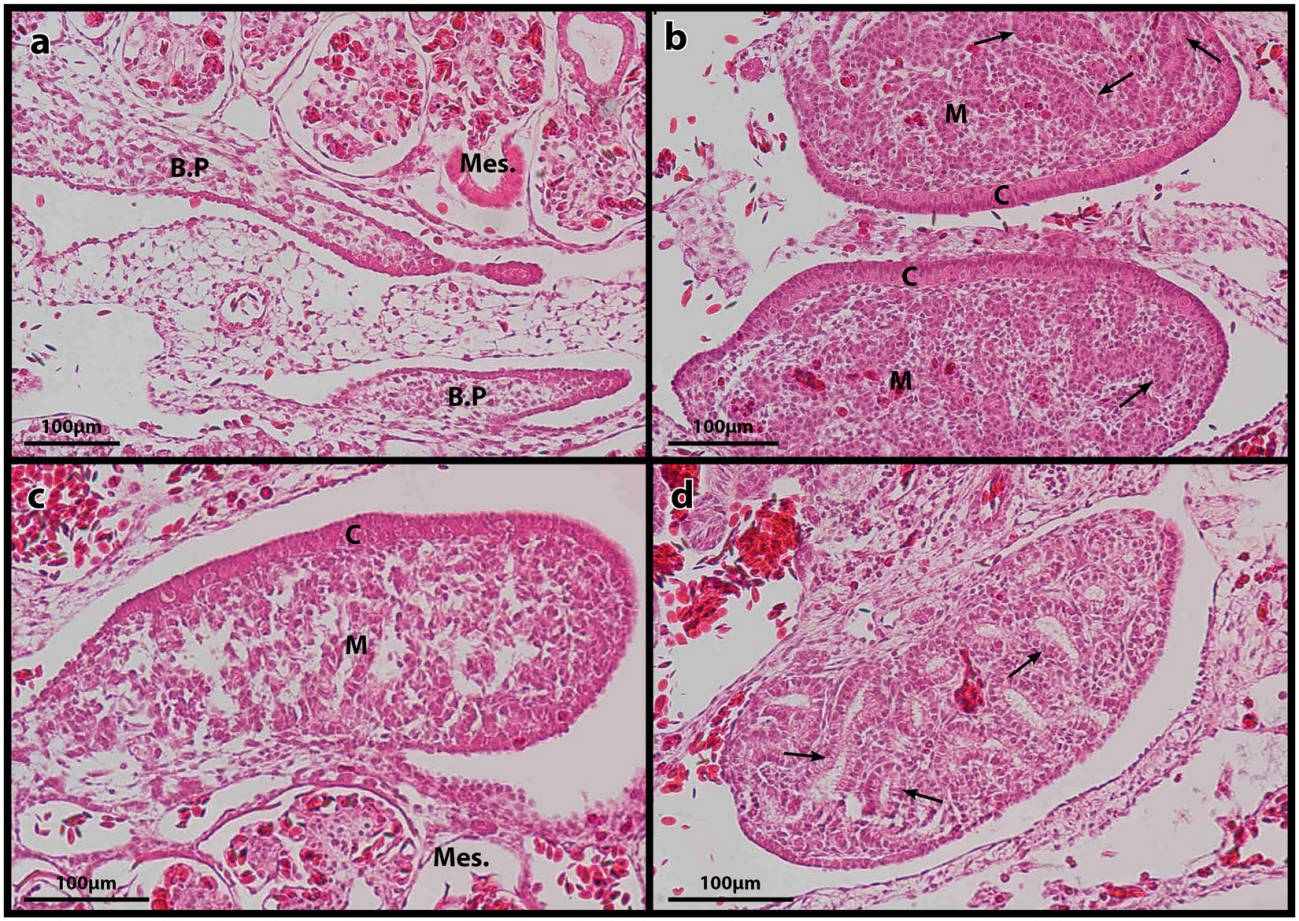
Histological sections of embryonic *Pogona vitticeps* urogenital systems stained with haematoxylin and eosin (H & E). (**a**) Bipotential gonads with developing cortex and medullary regions during migration towards the anterior mesonephros. (**b**) Ovotestes from an embryo incubated at 36 °C undergoing sex reversal showing a proliferating cortex with oogonia, a medulla with numerous rudimentary seminiferous tubules. (**c**) Differentiated ovary with a reducing medulla, cortex proliferating with oogonia. (**d**) Differentiated testes with a reducing cortex, and medulla with developing seminiferous tubules. B. P = bipotential gonad, Mes. = mesonephros, C = cortex, M = medulla, black arrows = seminiferous tubules.

**Figure 3:**
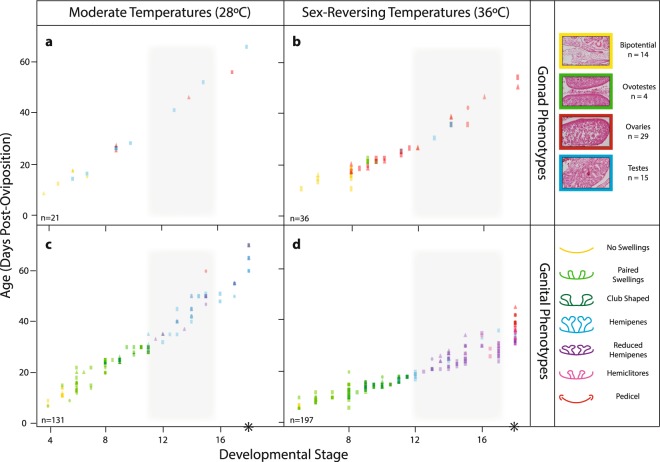
Timing of gonad (**a**,**b**) and genital (**c**,**d**) development for *Pogona vitticeps* at normal (28 °C; **a**,**c**) and sex-reversing (36 °C; **b**,**d**) incubation temperatures. Sexual phenotype is indicated by colour as per the legend, and sexual genotype is indicated by shape (squares = ZZ specimens, triangles = ZW specimens, circles = unknown). The grey shading defines the period of temporary pseudohermaphroditism during female development, persisting for approximately 9 stages. The black asterisks denote approximate time of hatching (stage 18, ~73 dpo at 28 °C and ~47 dpo at 36 °C; Holleley *et al*.^19^). Genital development data was re-analysed from Whiteley *et al*.^27^.

Early in development (approximately stages 4 to 8), the bipotential gonads were loosely attached to the posterior end of the mesonephros and exhibited an elongated shape as they progressively moved to an anterior position (Fig. 2a). Gonad-mesonephric attachment increased once the gonads were at the anterior-most portion of the mesonephros, and the gonads developed a rounder shape. Defined cortex and medullary layers were present just before gonad differentiation. This process was observed in all specimens regardless of genotype (ZZ or ZW), and whether or not they underwent sex reversal. Testes differentiation occurred by approximately stage 9 (Figs 2d and 3a) and was characterised by reduction of the cortex and proliferation of the medulla, within which seminiferous tubules formed (Fig. 2d). Two ZZm offspring of sex reversed mothers incubated at 28 °C exhibited differentiated testes at stages 6 and 7 respectively (Fig. 3a), which is earlier than what was observed in ZZm offspring of concordant mothers (ZW). Ovarian differentiation (begins approximately stage 8; Fig. 3a,b) was characterised by a reduced medulla and a proliferating cortex with oogonia (Fig. 2c).

### Asynchronous internal and external female sexual development

Regardless of whether sex was determined by genotype or temperature, the development of sexual phenotypes (genitalia described in Whiteley *et al*.^27^; gonads, and accessory ducts; Fig. S1) in female *P. vitticeps* followed the same pattern described for other squamates. However, there was a delay in relative timing of gonad and genital differentiation in both concordant (ZW) and sex reversed (ZZf) females. Ovaries began differentiating at stage 8 (Fig. 3a,b), whereas the genitalia continued to masculinise until distinctly bilobed hemipenes formed, typically by stage 11 (Fig. 3c,d). Later in development (approximately stages 14–15), long after ovarian differentiation, the mature female genital phenotype (pedicel) began to develop as the hemipenes regressed (Fig. 3c,d). This period of asynchronous development was characteristic of a temporary pseudohermaphroditism phase, or TPH (grey shading Fig. 3). We estimated the TPH phase to occur from stage 8 to stage 15, equating to approximately 45% of total development, with some inter-individual variation in the timing of events (see Figs 1, 3, and File S1).

### Sex reversal specific occurrence of ovotestes

We observed ovotestes (Fig. 2b) in 4 of 10 ZZf individuals exposed to sex-reversing temperatures (36 °C) at stages 9–9.5 of development (Fig. 2b). The occurrence of ovotestes coincided with the transition from bipotential to committed gonad. Ovotestes were characterised by a typical ovarian cortex with oogonia. However, instead of the medulla consisting of loose, randomly arranged connective tissues, the cells had condensed into rudimentary seminiferous tubules, occasionally with lumen, akin those seen in normal testes. We did not observe ovotestes in ZW individuals incubated at 36 °C at this stage (4 individuals), or in ZZ or ZW individuals incubated at 28 °C (5 and 3 individuals respectively).

### Hemipenal ultrastructures

**Figure 4:**
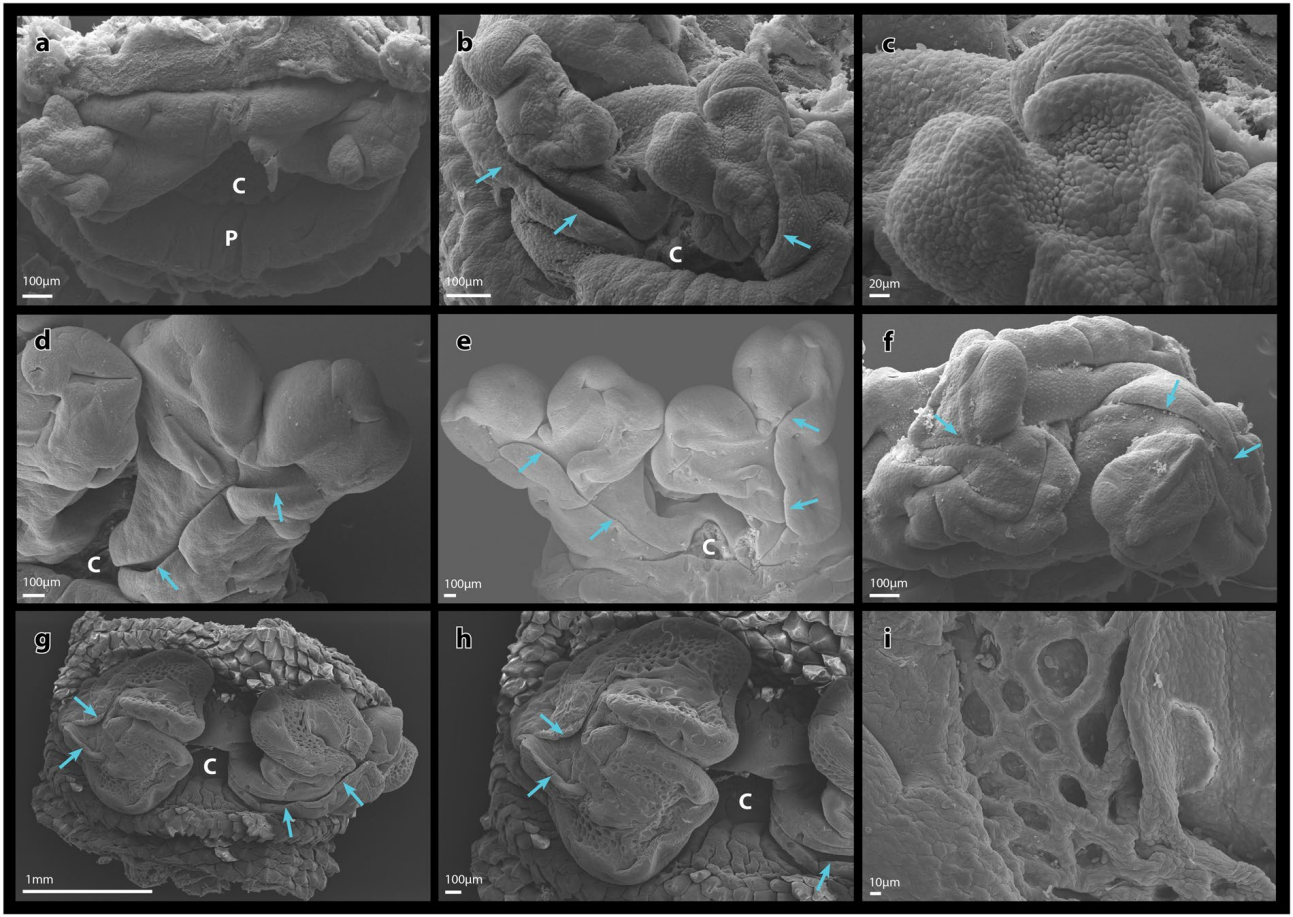
Homologous hemipenal structures in male and female *Pogona vitticeps*. Scanning electron micrographs of stage 14 (**a**–**f**) and stage 17 (**g**–**i**) embryonic genitalia. (**a**) Reduced hemipenes of a ZW female showing trilobes. (**b**) ZW female from the same clutch as specimen in with well-developed bilobed hemipenes each with a sulcus spermaticus. (**c**) Enhanced view of right hemipenis of specimen in (**b**) showing the beginnings of the hemipenal lattice. (**d**) Bilobed hemipenes of a ZZ male with a sulcus spermaticus. (**e**) Bilobed hemipenes with sulcus spermaticus of a ZZ embryo incubated at 36 °C that did not undergo sex reversal. (**f**) Bilobed hemipenes with sulcus spermaticus of a sex reversed female. (**g**) Well developed bilobed hemipenes of a ZZ male showing the sulcus spermaticus and hemipenal lattice. (**h**) Enhanced view of left hemipenis of specimen shown in (**g**). (**i**) Enhanced view of the hemipenal lattice from specimen shown in (g) and (h). C = cloaca, P = pedicel, blue arrows = sulcus spermaticus. The specimens presented in E and F were validated histologically to have testes and ovaries respectively.

Morphological comparison of genital phenotypes between males (ZZm), concordant (ZW), and sex reversed females (ZZf) at stage 14 using SEM showed conserved ultrastructural characteristics. The hemipenes of the ZW (Fig. 4b,c) and the ZZf female (Fig. 4f) were very similar, and both exhibited a sulcus spermaticus, which extended from the base of each hemipenis, and bifurcated at the bilobes. The hemipenes of two ZZ males (Fig. 4d,e) were more similar to each other than they were to those of the ZW and ZZf females as they displayed a more uniformly shaped sulcus spermaticus and the hemipenes exhibited a smooth surface interspersed with irregular invaginations (Fig. 4b–f). A late stage 17 ZZm male examined (Fig. 4g–i) exhibited a distinctive ultrastructure we have termed the hemipenal lattice. This structure comprised of a furrow that extended along the distal surface of each lobe of the hemipenes, within which was a series of interconnected indentations forming a lattice-like structure (Fig. 4g–i).

Some variability in the timing of hemipenis regression was observed in stage 14 ZW females. Two specimens from the same clutch exhibited differing phenotypes; one female had begun hemipenis regression with each appendage exhibiting a trilobed appearance (Fig. 4a), while the other female possessed well-formed bilobed hemipenes with a sulcus spermaticus (Fig. 4b,c). There was also a texture on the surface of the hemipenes suggestive of a precursor to the hemipenal lattice observed in the late stage male (Fig. 4b).

## Discussion

Our results show that late regression of hemipenes in female *Pogona vitticeps* is part of a prolonged period of temporary pseudohermaphroditism (TPH) during which ovaries and hemipenes are both present (Figs 1 and 3). This TPH phase was observed in both genetically concordant (ZW) and sex-reversed females (ZZf). Consistent with the other two species of squamates displaying TPH, all males (ZZm) of *P. vitticeps* displayed a fast succession of gonad development and hemipenis differentiation, as is also the case in most other amniotes that have been studied^17,18,34–36^.

The TPH phase in female development of *Pogona vitticeps* is the third case of TPH in squamates, out of four species that have been examined to date^30–32^. This raises the possibility that TPH might be relatively common in squamates, and that gonad differentiation and genital development are much less tightly linked than currently thought. A phylogenetically widespread TPH phase (Fig. 1) may also explain why many squamate species display very limited sexual dimorphism at hatching^37,38^. This further raises the possibility that embryonic or hatchling sex identification in squamates using only genitalia may be incorrect if TPH is not recognised. In addition, the variable timing and duration of the TPH phase hints at an evolutionarily flexible relationship between ovary differentiation and genital formation (Fig. 1). This might be a contributing factor to the very fast evolution of squamate genital morphology^34^, and may explain the evolution of extreme female phenotypes, such as the strongly developed hemipenes seen in females of two snake species, *Pseudoficimia frontalis*^29,39^, and *Bothrops insularis*^40^. Thus, a better understanding of the frequency of TPH among squamates, and the mechanisms by which genital development is governed by the ovaries, has the potential to provide a substantial advance in the understanding of squamate (and possibly amniote) sexual evolution. It is also interesting to speculate whether TPH is a trait associated with thermosensitive sex determination, since the only species not to display a prolonged TPH phase (*A. carolinensis*) is also the only strictly GSD species where this has been studied. While the sex determination mode of *B. imbricata* is unknown, the only other species in the same family (Anguidae) that has been investigated (the southern alligator lizard, *Gerrhonotus multicarinatus*) may be GSD with a thermal influence^38,41^. We have further demonstrated that in GSD species with known thermosensitivity, TPH is associated with both male and female heterogamety (ZW/ZZ system *of P. vitticeps;* XY/XX of *N. ocellatus*) (Fig. 1)^24,42^.

It is unclear what mechanisms are behind the asynchronous internal and external sexual development in squamate TPH, but hormone-related processes likely play an important role. In particular, delayed hormonal secretion could be a main factor in determining TPH of *P. vitticeps*, as a recent study showed that the estrogen inhibitor fadrozole prevents the hemipenes of *P. vitticeps* from regressing until after hatching^43^, suggesting that estrogen is required for the formation of the female genital phenotype. Artificial introduction of testosterone in adult female leopard geckoes (*Eublepharis macularius*) induces the formation of hemipenes, demonstrating that the genitalia can be sensitive to endogenous hormones^44^. These findings are consistent with research demonstrating that the gonads of some lacertilians gain the ability to synthesize sex steroids only after hatching^45–47^. It is also possible that TPH may be due to a delay in hormone receptiveness of the genitalia. While little is known about this in squamates, in the one species studied that does not exhibit TPH (*Anolis carolinensis*), steroid hormone receptors are expressed dimorphically during external genital development, such that embryonic hemipenes express more androgen receptors and female hemiclitores express more estrogen receptors^48,49^. This might indicate that genital tissues are indeed able to display differential receptiveness to hormones throughout development, adding another layer of complexity to the sexual development of squamates.

In addition to asynchronous gonadal and genital sexual development, we also observed a relatively high frequency of ovotestes during early gonad differentiation in genetically male individuals at sex-reversing temperatures (4 out of 10 of ZZf specimens at stages 9–9.5 at 36 °C). Naturally occurring ovotestes have only been observed in reptiles in isolated cases^50,51^. However, they have been experimentally induced in one lizard and several turtles with TSD that were incubated at their pivotal temperature (the temperature at which the sex ratio is approximately 50:50) or at fluctuating temperature regimes^52–55^. This has led to the suggestion that ovotestes arise in situations where the levels of estrogen and testosterone are similar and acting antagonistically^52^. However, ovotestes can also be indicative of an epigenetic re-programming event, causing the transition from one sex to another, as has been observed in some sequentially hermaphroditic fish^56,57^. Additionally, new single cell sequencing techniques have shown that despite apparently committing to a fate, cells can change trajectories during development, suggesting that gonadal differentiation may be quite flexible^58,59^. Ovotestes in *P. vitticeps* may be triggered by the initiation of environmental sex reversal via the activation of chromatin modifying genes, allowing ovotestes to develop as a result of simultaneous and competing testicular and ovarian tissue hormonal activity during sex reversal^9,11,57,60,61^. It may be that ovotestes are more broadly indicative of environmental effects on sex determination, and may facilitate the identification of new thermally sensitive species.

Finally, it is interesting that in all three known cases of sexual development asynchrony, the TPH phase is displayed by females only. Even in *A. carolinensis*, which does not exhibit a prolonged TPH phase, the hemipenes regress quickly (within half a stage) to form the female hemiclitores^29,32,48^. The hemipenes of female *P. vitticeps* resemble those of males in every respect, including the general ultrastructure and the presence of a male-specific functional character (sulcus spermaticus). This is consistent with previous suggestions that female genital phenotypes of amniotes arose through a hormonal modification of the male phallus^27,49,62^.

This study highlights the need to better understand the nuanced influences of temperature on the development of thermally sensitive species, such as *P. vitticeps*. This is particularly important given that increasing global temperatures can destabilise population sex ratios in temperature sensitive species worldwide^63–67^. Thermosensitive sex determination systems, which are being found to be increasingly common, may face additional challenges in a rapidly changing climate^20,68^. We have also highlighted that a tendency to rely on insufficiently tested assumptions, and a bias towards research on male phenotypes, has resulted in an incomplete view of female squamate sexual development. Further research on the interplay between genetic, hormonal, and environmental determinants of squamate development have the potential to reveal a more complete understanding of the drivers of squamate sexual evolution.

## Methods

### Embryo sampling

Animal breeding and embryo sampling for the embryological staging series is described in Whiteley *et al*.^27^. Briefly, embryos from combinations of high (36 °C) and low (28 °C) incubation temperatures, and genotypes (ZZm, ZZf, ZW) were examined. 296 eggs were obtained from the breeding colony established at the University of Canberra, while an additional 33 eggs were obtained from a commercial breeder. The breeding colony contains a mix of wild caught and lab bred adults of known sex (validated by genotyping and hemipenal eversion; File S1). The sex chromosome complement of all specimens was determined using a sex specific PCR test, with DNA extracted from embryonic blood sampled from the interior of the eggshell, as described previously^27^.

### Histology

To determine the timing of gonad differentiation at the two incubation temperatures (36 °C and 28 °C), specimens were staged according to the system presented in Whiteley *et al*.^27^ and sampled at three periods throughout development; early (stages 4–8.5, n = 21), middle (stages 9–12, n = 25), and late (stages 13 onwards, n = 16). Once a mature gonadal phenotype was consistently observed, sampling intensity was lowered. A total of 62 specimens were examined histologically (21 at 28 °C and 41 at 36 °C). The larger sample sizes at sex reversing temperatures ensured sufficient data were collected for morphological changes during sex reversal (File S1).

Specimens were processed following standard histological procedures^69^. Briefly, the urogenital systems were dehydrated through graduations of ethanol (70%, 90%, 100%) and two changes of xylene for 45 minutes each, before being embedded in paraffin wax, and sectioned 6 µm thick using a Leica Rotary Microtome (Leica Microsystems Pty Ltd, Waverley, Australia). The slides were stained with Meyer’s haematoxylin and eosin (H & E), with a staining time of three minutes in haematoxylin, and 10 dips in 0.25% eosin in 80% ethanol, before being mounted in depex. All slides were analysed using standard light microscopes, and the gonads and accessory ducts were defined using established cellular characteristics described previously for other reptile species^16,18,45,52^. Detail regarding the development of the accessory ducts is provided in the supplement (Fig. S1).

### Scanning electron microscopy

We investigated the degree of homology between the genital structures of males (ZZm), and concordant (ZW) and sex reversed (ZZf) females at the same developmental stage using scanning electron microscopy (SEM). A total of 36 ZW female offspring were incubated at either 28 °C (25 specimens) or 36 °C (11 specimens) and sampled at the same developmental stage (stage 14) across treatments (see File S1). An additional stage 17 male (ZZm) was examined to assess the later structural development of the hemipenes. Specimens were genotyped for the sex specific marker (see above) and a subset were processed for SEM using standard techniques. Briefly, after formalin preservation, the whole genitalia were dissected, dehydrated through graduations of ethanol (70%, 90%, 100%). They were critical point dried and coated with gold according to the manufacturer’s instructions and imaged using a Zeiss EVO LS 15 (Carl Zeiss Pty Ltd, North Ryde, Australia). The late stage 17 ZZm male was critical point dried using hexamethyldisilazane and coated with a 10 nm thick layer of iridium and imaged using a Field Emission Scanning Electron Microscope JEOL JSM-7001 F (JOEL Australasia Pty Ltd, Frenchs Forest, Australia). Sex reversal in ZZ specimens incubated at 36 °C was validated using gonadal histology following the methods described above.

### Ethics approval

All experimental protocols were conducted with the permission of Animal Ethics Committees at the University of Canberra (CEAE15-21) and the University of Queensland (SBS/295/16). All experiments were conducted in accordance with guidelines and regulations established by these committees.

## Electronic supplementary material

Supplementary Information

File S1

## Data Availability

All data generated and analysed in this study are available in Supplementary File S1. Registered voucher specimens for each *Pogona vitticeps* embryonic stage are available for inspection or inter-institutional loan from the Australian National Wildlife Collection, CSIRO (Registration numbers R11229 – R11246).
