# Supplementary material for: Developmental asynchrony and antagonism of sex determination pathways in a lizard with temperature-induced sex reversal
Source: Sci Rep. 2018 Oct 5;8:14892. doi: 10.1038/s41598-018-33170-y (PMC6173690; doi:10.1038/s41598-018-33170-y)
Supplement: Supplementary file 1 — Supplementary Information [file 41598_2018_33170_MOESM1_ESM.pdf]

Developmental asynchrony and antagonism of sex determination pathways in a lizard with temperature-induced sex reversal

Sarah L. Whiteley[1,2,3]

Vera Weisbecker[3]*

Arthur Georges[1]

Arnault Roger Gaston Gauthier[4]

Darryl L. Whitehead[4]

Clare E. Holleley[1,2]*

*corresponding

ORCID IDs

Sarah L. Whiteley: 0000-0003-3372-4366

Vera Weisbecker: 0000-0003-2370-4046

Arthur Georges: 0000-0003-2428-0361

Arnault R. G. Gauthier: 0000-0003-2467-6016

Clare E. Holleley: 0000-0002-5257-0019

*Correspondence: clare.holleley@csiro.au; v.weisbecker@uq.edu.au;

[1] Institute for Applied Ecology, University of Canberra, Canberra, ACT, Australia

[2] Australian National Wildlife Collection, National Research Collections Australia, CSIRO, Canberra, ACT, Australia

[3] School of Biological Sciences, University of Queensland, Brisbane, QLD, Australia

[4] School of Biomedical Sciences, University of Queensland, Brisbane, QLD, Australia

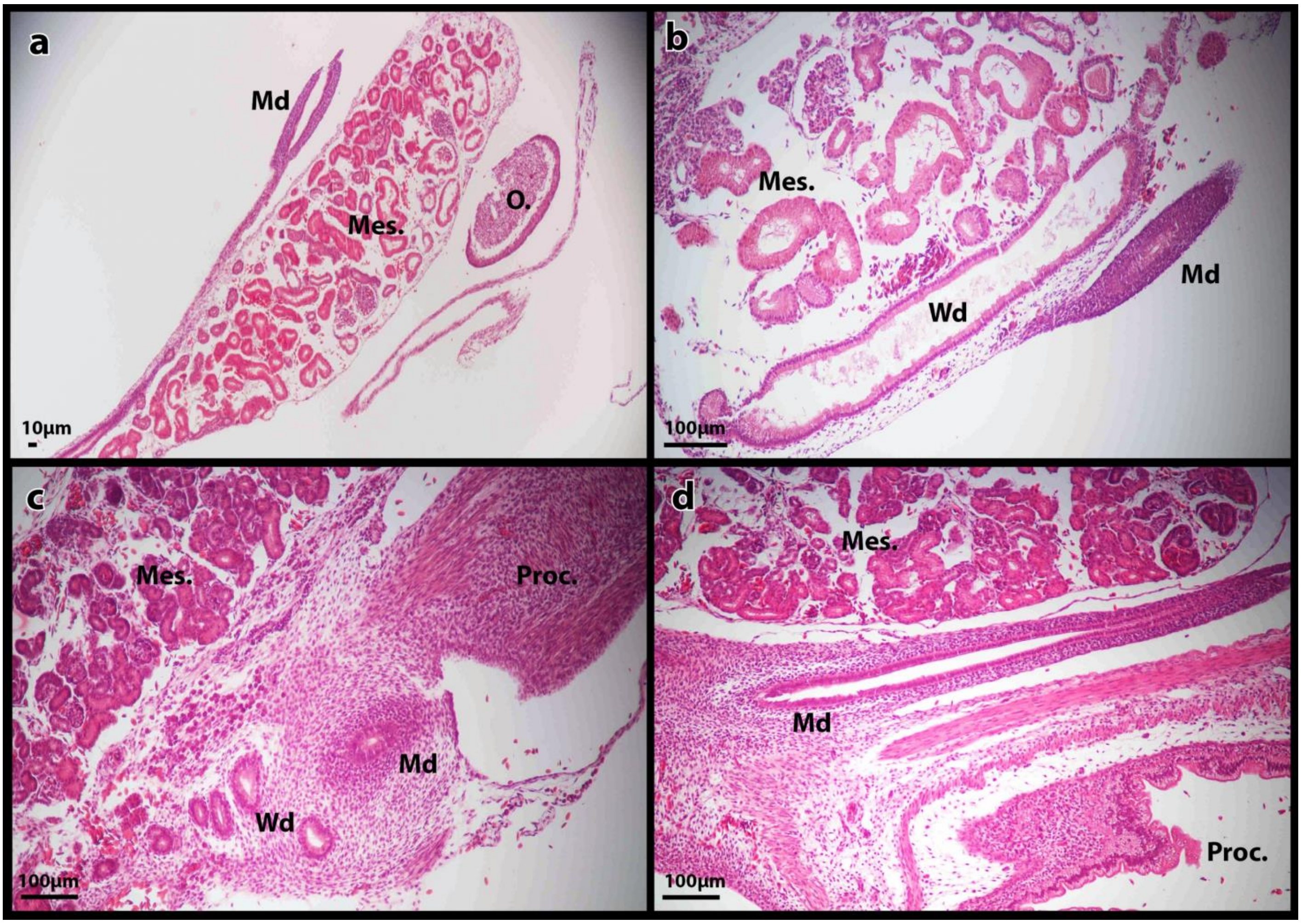

Figure S1: Histological sections of embryonic *Pogona vitticeps* urogenital system stained with haematoxylin and eosin (H & E). a) Left mesonephros with a Müllerian duct extending along its entire length, and an ovary with clear cortex and medulla differentiation. They are characterised as having dense, darkly stained circles with a lumen surrounded by epithelial cells and mesenchymal cells separated by basal lamina. b) A Wolffian duct comprised of simple cuboidal epithelium with extensive lumen within the outer layer of epithelial cells surrounding the mesonephros, to which a Müllerian duct is attached. c) Müllerian and Wolffian tubules embedded within the connective tissues below the proctodaeum and beside the metanephros. d) Posterior end of a Müllerian duct embedded within the connective tissues between the proctodaeum and mesonephros. Mes. = Mesonephros, Md = Müllerian duct, Wd = Wolffian duct, O = ovary, Proc. = proctodaeum.
